# Adsorption of Sulfonamides in Aqueous Solution on Reusable Coconut-Shell Biochar Modified by Alkaline Activation and Magnetization

**DOI:** 10.3389/fchem.2021.814647

**Published:** 2022-01-21

**Authors:** Ying Sun, Lili Zheng, Xiaoyan Zheng, Dao Xiao, Yang Yang, Zhengke Zhang, Binling Ai, Zhanwu Sheng

**Affiliations:** ^1^ Haikou Experimental Station, Chinese Academy of Tropical Agricultural Sciences, Haikou, China; ^2^ College of Food Science and Engineering, Hainan University, Haikou, China; ^3^ Haikou Key Laboratory of Banana Biology, Haikou, China

**Keywords:** biochar, coconut shell, sulfonamide antibiotics, adsorption, modification

## Abstract

Biochar is a low-cost adsorbent for sorptive removal of antibiotics from wastewater, but the adsorption efficiency needs to be improved. In this study, coconut-shell biochar was activated with KOH to improve the adsorption efficiency and magnetically modified with FeCl_3_ to enable recycling. The amount of KOH and the concentration of FeCl_3_ were optimized to reduce the pollution and production cost. The KOH-activated and FeCl_3_-magnetized biochar gave good sulfonamide antibiotic (SA) removal. The maximum adsorption capacities for sulfadiazine, sulfamethazine and sulfamethoxazole were 294.12, 400.00 and 454.55 mg g^−1^, respectively, i.e., five to seven times higher than those achieved with raw biochar. More than 80% of the adsorption capacity was retained after three consecutive adsorption-desorption cycles. A combination of scanning electron microscopy, Brunauer-Emmett-Teller analysis, X-ray diffraction, Fourier-transform infrared and Raman spectroscopies, and magnetic hysteresis analysis showed that KOH activation increased the specific surface area, porosity, and number of oxygen-rich functional groups. Iron oxide particles, which were formed by FeCl_3_ magnetization, covered the biochar surface. The SAs were adsorbed on the modified biochar via hydrogen bonds between SA molecules and -OH/-COOH groups in the biochar. Investigation of the adsorption kinetics and isotherms showed that the adsorption process follows a pseudo-second-order kinetic model and a monolayer adsorption mechanism. The adsorption capacity at low pH was relatively high because of a combination of π^+^-π electron-donor-acceptor, charge-assisted hydrogen-bonding, electrostatic, and Lewis acid-base interactions, pore filling, van der Waals forces and hydrophobic interactions. The results of this study show that magnetically modified biochar has potential applications as an effective, recyclable adsorbent for antibiotic removal during wastewater treatment.

## 1 Introduction

Antibiotics are widely used to treat various diseases of humans and animals (Mandu et al., 2015; Ying et al., 2017). However, about 70–90% of antibiotics are excreted in feces and urine. Environmental degradation of excreted antibiotics is difficult and they are widely found in soil, surface water, and groundwater (Cheng et al., 2018; Huang et al., 2019). Although the concentrations of residual antibiotics in the environment are low (nanograms to milligrams per liter), they cause increases in the drug resistance of pathogens (Reguyal and Sarmah, 2017), and the development of antibiotic-resistant bacteria and antibiotic-resistance genes (Martínez, 2008). They therefore adversely affect human health and ecosystems. This problem has aroused widespread concern and the development of economical and effective methods for the removal of antibiotics from wastewater and soil is important.

Many methods have been developed for antibiotic removal from water, e.g., adsorption (Moussavi et al., 2013), reverse osmosis (Košutić et al., 2007), photolysis, chemical oxidation (Dai et al., 2020), and biodegradation (Gadipelly et al., 2014). Adsorption is a convenient, effective, and environmentally friendly method (Patrycja et al., 2019). Commonly used adsorbents include graphene (Gao et al., 2012), activated carbon (Ndagijimana et al., 2019), carbon nanotubes (Anjum et al., 2019), metal-organic frameworks (Chen X et al., 2019), mineral materials (Wu et al., 2019), and biochar (Wang and Wang, 2019). Supplementary Table S1 presents the comparison of maximum adsorption for sulfonamide antibiotics on various activated carbon materials reported in previous studies. Supplementary Table S2 presents various modified adsorbents for sulfonamides removal. Among these, biochar has attracted wide attention for the removal of pollutants from soil and water. Biochar is a carbonaceous porous material (Patrycja et al., 2019); it is the product of the pyrolysis of biomass (plant raw materials or municipal waste) under low-oxygen or non-oxygen conditions (Liu et al., 2015). Biochar is a low-cost adsorbent, and its surface functional groups, e.g., phenolic hydroxyl, condensed aromatic rings, ether, and carboxyl (Liu et al., 2015), provide active sites for antibiotic adsorption. However, the specific surface area and pore size of the initial biochar are limited, therefore further modification is needed to increase its adsorption capacity (Cheng et al., 2020). Modification methods include treatments with acids, alkalis, oxidants, and metal salts or metal oxides, and carbonaceous material modification (Zhu et al., 2018; Wang and Wang, 2019). The adsorption migration channels and adsorption capacities of alkali-treated biochar are larger than those of char obtained by simple pyrolysis because carbon loss during alkaline activation expands the micropores to mesopores (Luo et al., 2016). However, due to the very fine particles and low density of KOH activated biochar, after mixing with a solution, it is difficult to separate the biochar for recycling. Compared with traditional activated biochar, it is easier to separate and recycle magnetic activated biochar by using external magnetic fields (C et al., 2018). Moreover, the large-scale use of biochar in wastewater treatment plants has been limited due to biochar variability and small particle size, slowing flowrates and causing pressure drops in columns. Filtration steps would be slow, which raised the operation cost. Magnetic removal attenuates these issues, improves removal efficiency and allows smaller particle size biochar to be recovered from batch (stirred tank) processes (D. et al., 2018; Dewage et al., 2019). Compared with chemical coprecipitation, iron impregnation is a simpler and more economical magnetization method (Zhang X et al., 2020).

Hainan Island is referred to as “Coconut Island”, and its coconut production accounts for about 80% of China’s coconut-planting area and output. Coconut shell accounts for about 85% of the fruit weight and improper disposal causes environmental problems and is a waste of resources (Gonzaga et al., 2018). Conversion to biochar provides an alternative strategy for coconut-shell treatment.

According to our preliminary experiment, among biochars prepared from coconut shell, *Camellia oleifera* shell, cassava stalks, rubber wood blocks, and banana stalks, coconut-shell biochar has the highest capacity for sulfonamide adsorption in aqueous solution (Supplementary Figure S1A). In this study, recyclable biochar was obtained by modifying coconut-shell biochar via KOH activation and FeCl_3_ magnetization, and used for adsorption of sulfonamide antibiotics (SAs). Scanning electron microscopy (SEM), the Brunauer-Emmett-Teller (BET) method, X-ray diffraction (XRD), Fourier-transform infrared (FTIR) and Raman spectroscopies, and magnetic hysteresis analysis were used to characterize the adsorbents. The adsorption behaviors of SAs, i.e., the adsorption kinetics, isotherms, and adsorption mechanisms, and magnetic biochar regeneration were also investigated.

## 2 Materials and Methods

### 2.1 Biochar Preparation and KOH Activation

Coconut shell was obtained from Hainan, China. The coconut shell was washed with ultra-pure water and dried in an oven at 80°C for 24 h. After milling and passing through a 100-mesh sieve, the coconut-shell powder was pyrolyzed under N_2_ in a muffle furnace at 700°C at a heating rate of 10°C min^−1^ for 1 h; this sample was denoted by BC. The coconut shell powder pyrolyzed under N_2_ to 400°C was mixed with KOH at mass ratios of 1:1, 1:1.5, 1:2, 1:2.5, 1:3, 1:3.5, 1:4, 1:4.5, 1:5, and 1:5.5, the samples were pyrolyzed under N_2_ at 700°C at a rate of 10°C min^−1^ for 1 h. After cooling, the solids were washed with 35% HNO_3_ to remove impurities, washed with ultra-pure water to stabilize the pH, and dried in an oven at 105°C for 5 h. The KOH-activated samples were denoted by BC-KOH_*k*_ (*k* = 1, 1.5, 2, 2.5, 3, 3.5, 4, 4.5, 5, and 5.5).

### 2.2 Magnetic Modification of KOH-Activated Biochar

The BC-KOH_*k*_ sample with the highest adsorption capacity was mixed with FeCl_3_·6H_2_O solution at concentrations of 25, 50, 75 and 100 mmol L^−1^ at a ratio of 1:100 (w/v); the mixtures were shaken at 150 rpm for 5 h to complete impregnation, and then dried in an oven at 105°C overnight. The magnetic biochar, which was denoted by *n*MBC-KOH_*k*_ (*n* = 25, 50, 75 and 100) was obtained by pyrolysis under N_2_ at 700°C for 1 h in a muffle furnace at a heating rate of 10°C min^−1^.

### 2.3 SA Determination

SA samples were analyzed by ultra-high-performance liquid chromatography (UPLC). A UPLC-PDA system (Waters ACQUITY UPLC M-Class) equipped with an ACQUITY UPLC BEH C18 column (2.1 × 150 mm, 1.7 μm) was used. Phosphoric acid (0.05 mol L^−1^) and methanol solution was selected as mobile phases A and B; the elution procedure is shown in Supplementary Table S3. The injection volume was 10 μl, the flow rate was 0.3 ml min^−1^, and separation was performed at 30°C. The detection wavelengths for sulfadiazine (SDZ), sulfamethazine (SMT), and sulfamethoxazole (SMX) were 265, 243, and 268 nm, respectively. The total running time of the system was 8 min. In the SA concentration range, the correlation coefficient (*R* ^2^) of the standard curve after calibration was more than 0.99.

### 2.4 Characterization of Adsorbents

The surface morphologies of the adsorbents under 5 kV ultra-high pressure (EHT) were studied by field-emission SEM (Zeiss Merlin, Germany). The C, H and N contents were determined by using an elemental analyzer (VARIO EL III, Elementar Inc., Germany). The O content was calculated as the total weight of biochar minus the C, H, N, and ash contents. A BET analyzer (Quantachrome Autosorb IQ, United States) was used to determine the specific surface area, total pore volume, and average pore size (*D*_p_) of the biochar from N_2_ adsorption-desorption isotherms at 77 K. The specific surface area of the adsorbent was calculated by BET and density functional theory methods, and the mesopore volume was calculated by the Barrett-Joyner-Halenda method. The surface functional groups of the biochar were identified by performing FTIR spectroscopy (FTS6000, Bio-Rad, California, United States) in the range 400–4000 cm^−1^ with a resolution of 4 cm^−1^. The Zeta potential of the magnetic biochar was measured with a Zetasizer Nano analyzer (Malvern Instruments Inc., United Kingdom) in the pH range 3.00–10.00. Raman spectroscopy (Renishaw in Via Raman spectrometer, Gloucestershire, United Kingdom) was used to analyze the carbon structure of the adsorbent. The magnetic properties of the samples were investigated with a SQUID-VSM magnetic property measurement system (VSM, PPMS-9, Quantum, United States); the hysteresis curves were recorded at 300 K. The mineral phases and crystal structures of the samples were identified by XRD (Bruker D8 Advanced diffractometer, Cu Kα radiation). The scanning range was 10°–80° (2*θ*) and the scanning speed was 0.4°min^−1^.

### 2.5 Adsorption Experiments

Stock solutions of SDZ, SMT, and SMX were prepared in ultra-pure water with 0.1% methanol (a cosolvent effect was not observed). The pH was not adjusted. The removals of SAs by BC, BC-KOH_*k*_, and *n*MBC-KOH_*k*_ were evaluated by performing adsorption experiments with SDZ, SMT, and SMX. A SA stock solution was used to prepare a 50 mg L^−1^ reaction solution, with 0.01 mol L^−1^ NaCl as the background electrolyte solution to maintain constant ionic strength. The solution pH was adjusted to 5 ± 0.10 with 0.01 mol L^−1^ NaOH and 0.01 mol L^−1^ HCl solutions. The adsorbent (5 mg) and 50 mg L^−1^ SA solution (100 ml, solid: liquid ratio 1:20) were placed in a 250 ml Erlenmeyer flask, and then the flask was sealed; three parallel experiments were performed for each group. The flasks were placed in a constant-temperature water bath at 25°C and shaken at 150 rpm for 24 h. The mixture was filtered through a 0.22 μm poly (ether sulfone) water-based filter syringe, and then UPLC was used to determine the filtrate concentration.

### 2.6 Adsorption Kinetics

The kinetics of SA adsorption on the magnetic biochar was investigated. The reaction conditions were the same as those described in Section 2.4, and the reaction system was enlarged appropriately. Samples of the suspension were sucked out at different time intervals over 1–1440 min, and three repeats were selected for analysis. The filtrate was obtained by filtering with a 0.22 μm poly (ether sulfone) water system filter syringe, and then the filtrate concentration was determined by UPLC. Pseudo-first-order (PFO) and pseudo-second-order (PSO) models were used to clarify the mechanism of SA adsorption on the magnetic biochar.

### 2.7 Adsorption Isotherms

Adsorption isotherm experiments were performed at pH 5 ± 0.10 and 25°C; the SA concentration was kept within the range 10–100 mg L^−1^. Three parallel experiments were carried out in each group. The samples were shaken in a constant-temperature water-bath oscillator for 12 h (the time required to reach equilibrium, as determined from the kinetic experiments). The filtrate concentration was determined by UPLC. The relationship between the adsorbate concentration and adsorption capacity was investigated by using Langmuir and Freundlich adsorption isotherm models. These models are suitable for describing multi-layer adsorption on heterogeneous surfaces and monolayer adsorption on homogeneous surfaces, respectively.

### 2.8 Influences of Initial pH and Co-existing Ions on SAs Removal by Biochar

A pH range of 3.0–10.0 was used to evaluate the effect of initial solution pH on the removal of SAs (50 mg L^−1^) with a solid: liquid ratio of 1:20. The solution was shaken at 150 rpm at 25°C in a constant-temperature water-bath oscillator for 12 h. Three parallel experiments were carried out in each group. Five ions, SO_4_^2−^, Cl^−^, NO_3_^−^, PO_4_^3−^, and NH_4_^+^, were selected as representative ions in real water (Zhang R et al., 2020; Dong et al., 2022). To evaluate the effects of ionic species/strength on the adsorption capacity of 50MBC-KOH_2.5_ to SAs, the ion concentration of 0–1 mmol L^−1^ was applied.

### 2.10 SAs Removal From Synthetic Wastewater

The adsorption by 50MBC-KOH_2.5_ of SAs in synthetic wastewater was also studied. The main components of the synthetic swine wastewater were glucose, NH_4_Cl, KH_2_PO_4_, MgSO_4_·7H_2_O and CaCl_2_·2H_2_O, at concentrations of 2830, 446, 132, 54 and 4 mg L^−1^, respectively (Cheng et al., 2020). The synthetic wastewater was added to stock solutions of SDZ, SMT and SMX to give final concentrations of 50 mg L^−1^. Using the same concentration of adsorbent (50 mg L^−1^), adsorption was performed at pH 5 ± 0.10, 25°C and 150 rpm for 24 h. A blank control sample (without biochar) was prepared for each experiment and used for evaluation by subtraction of the SA loss during the adsorption process. All experiments were repeated three times, and the average and standard deviation were recorded.

### 2.11 Recovery and Regeneration of Adsorbent

A SA solution (50 mg L^−1^) with a solid: liquid ratio of 1:20 and pH of 5 ± 0.10 was continuously shaken for 12 h at 25°C to reach complete adsorption equilibrium. The spent adsorbent was separated from the solution by vacuum filtration and dried at 80°C for 6 h. The dried spent adsorbent (30 mg) and 1.0 mol L^−1^ NaOH solution (300 ml) were added to a conical flask, and continuously shaken for 24 h in a constant-temperature water-bath shaker at 25°C to desorb the SAs on the spent adsorbent. After drying, the adsorbent was reused. Three cycles were performed.

## 3 Results and Discussion

### 3.1 KOH Activation and FeCl_3_ Magnetization

Due to KOH is a highly corrosive reagent and to ensure cost effectiveness, the effects of the ratio of KOH to coconut-shell biochar on the adsorption capacity were investigated. With the increase of KOH content, the adsorption capacity increased at first and then decreased, so a ratio of 2.5:1 was selected for biochar activation (Supplementary Figure S1B). It was found that the SA-adsorption capacity of biochar prepared at this ratio was much higher than values reported in the literature (Ahmed et al., 2017; Reguyal and Sarmah, 2017; Ying et al., 2017; Hao et al., 2018; Dewage et al., 2019; Huang et al., 2019; Wan et al., 2020; Zhang X et al., 2020), and the amount of KOH was nearly 40% lower than those reported in the literature (Cheng et al., 2020; Herath et al., 2020; Qu et al., 2021).

Magnetization was performed to enable biochar recovery and regeneration. The KOH-activated biochar modified with 50 mmol L^−1^ of FeCl_3_ gave the highest SA-adsorption capacity within the scope of selection (Supplementary Figure S1B). The comparison of SDZ, SMT and SMX adsorption capacity of BC, BC-KOH_2.5_ and 50MBC-KOH_2.5_ are showed in Supplementary Table S4. The total SAs adsorption capacity of the three kinds of biochar is 192.48, 1334.42 and 1074.28 mg g^−1^, respectively. After KOH activation (BC-KOH_2.5_), the adsorption capacity increased sharply, while magnetization (50MBC-KOH_2.5_) caused a decrease in adsorption capacity. Although the adsorption capacity of magnetization was reduced compared with KOH activation, it was more beneficial to the subsequent recycling. Hence, the subsequent adsorption experiments were carried out with 50MBC-KOH_2.5_ as the adsorbent.

The KOH activation process is as follows. 1) As the pyrolysis temperature increases, KOH gradually begins to crystallize, and thermal polymerization and curing take place on BC (Dai et al., 2019). Therefore, BC forms a porous structure, and KOH crystals are inserted and fixed in the pores. 2) When the pyrolysis temperature is further increased, the crystalline KOH particles gradually melt and react with BC to form K_2_CO_3_, K_2_O and K (Eqs 1–5) (Otowa et al., 1993; Pezoti et al., 2016; Fu et al., 2019; Prasannamedha et al., 2021). This further corrodes BC and increases the BET specific surface area, and the original mesopores and macropores gradually change into microporous structures (Anjum et al., 2019). 3) The CO, CO_2_, and other gases produced in step (2) are lost through the pores, which results in the formation of more micropores on the BC-KOH_2.5_ surface and a decreased carbon content.

Eqs 6–8 show that FeCl_3_ is first hydrolyzed and then converted to Fe_2_O_3_ during heating. With increasing temperature, CO and H_2_ are carbonized. At higher temperatures, CO can reduce Fe_2_O_3_ to Fe_3_O_4_, and this gives magnetic properties to the adsorbent (Dewage et al., 2019). Eqs 9–11 indicate the further reduction of carbon content after magnetization (Yang et al., 2016; Tang et al., 2018).

$$6KOH\;+\;2C\;\to \;2K\;+\;3H_{2}\;+\;2K_{2}CO_{3}$$ (1)

$$K_{2}CO_{3}\;+\;C\;\to \;K_{2}O\;+\;2CO$$ (2)

$$K_{2}O\;+\;C\;\to \;2K\;+\;CO$$ (3)

$$K_{2}CO_{3}\;\to \;K_{2}O\;+\;CO_{2}$$ (4)

$$2K\;+\;CO_{2}\;\to \;K_{2}O\;+\;2CO$$ (5)

$$FeCl_{3}\;+\;6H_{2}O\;\to \;Fe{\left(H_{2}O\right)}_{6}^{3+}\;+\;3Cl^{-}$$ (6)

$$2Fe{\left(H_{2}O\right)}_{6}^{3+\;}\to \;Fe_{2}O_{3}\;+\;6H^{+}\;+\;9H_{2}O$$ (7)

$$3Fe_{2}O_{3\;}+\;CO\;↔\;2Fe_{3}O_{4\;}+\;CO_{2}$$ (8)

$$FeCl_{3}\;+\;3H_{2}O\;\to \;Fe{\left(OH\right)}_{3}\;+\;3HCl$$ (9)

$$Fe{\left(OH\right)}_{3\;}\to \;FeO\left(OH\right)\;+\;H_{2}O$$ (10)

$$6FeO\left(OH\right)\;+\;4C\;\to \;2Fe_{3}O_{4}\;+\;4CO$$ (11)

### 3.2 SEM Analysis

**FIGURE 1:**
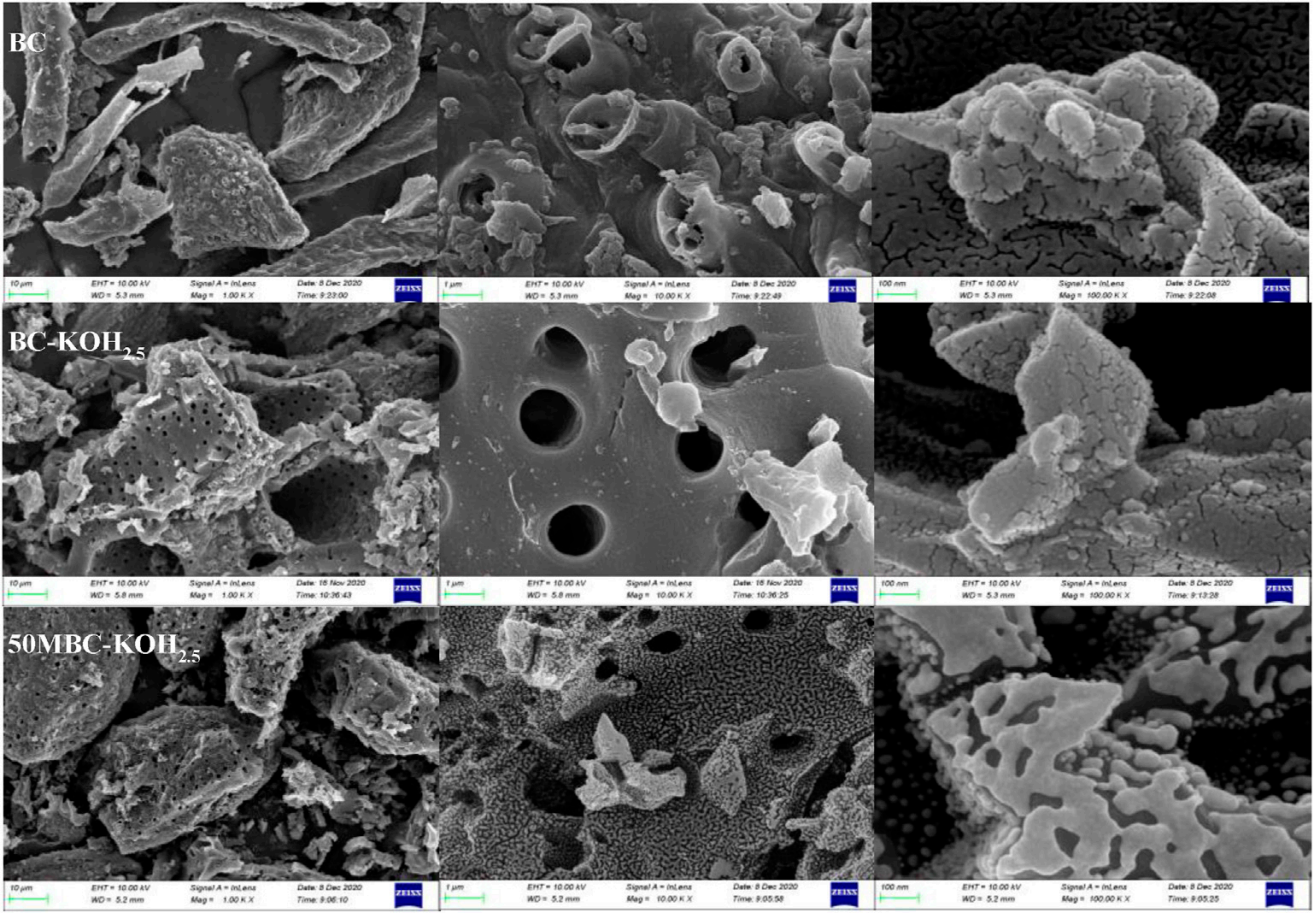
SEM images of BC, BC-KOH_2.5_ and 50MBC-KOH_2.5_.

Figure 1 shows the surface morphologies of BC, BC-KOH_2.5_ and 50MBC-KOH_2.5_. It can be seen that the BC surface is smooth and dense. There is a clear porous structure on the BC-KOH_2.5_ surface. This indicates increases in the surface area and total pore volume, which is consistent with the results reported by Yang B et al. (2019). This is because etching with alkali-metal compounds promotes the formation of micropores and macropores on biochar (Cheng et al., 2020). Magnetization does not significantly damage the porous structure, but the texture of the surface changes greatly; this may be caused by adhesion of iron oxides.

### 3.3 Elemental, BET Specific Surface Area and Pore Size Analysis

Table 1 showed the elemental analysis and textural characteristics of BC, BC-KOH_2.5_, 50MBC-KOH_2.5_ and coconut shell activated carbon. Elemental analysis showed that the weight percentage of carbon decreases after KOH activation. This is because of insertion of potassium ions into the coke, pore volume expansion, and carbon release in the form of CO_2_ and CO (Yang and Lua, 2003; Hui and Zaini, 2015). The increase in the oxygen weight percentage indicates an increased number of oxygen-containing functional groups after activation. This is consistent with the results reported by Herath et al. (2020). The increase in the nitrogen weight percentage can be attributed to residual nitric acid. The decreased carbon content after magnetization is due to further carbonization.

**TABLE 1:** Elemental analysis and textural characteristics of BC, BC-KOH_2.5_, 50MBC-KOH_2.5_ and coconut shell activated carbon (Ji et al., 2010).

Samples	Element analysis (wt%)				Textural characteristic		
	C	H	O	N	^a^S_BET_ (m^2^·g^−1^)	^b^V_t_ (cm^3^·g^−1^)	^c^V_mic_ (cm^3^·g^−1^)
BC	81.42	1.84	7.74	0	650.8	0.3804	0.302
BC-KOH_2.5_	77.72	1.47	20.04	0	1719.0	0.9070	0.648
50MBC-KOH_2.5_	62.67	1.10	19.98	0	1267.3	0.7095	0.464
^d^AC	90.86	-	8.13	1.01	624.0	0.3300	0.270

a S_BET_ - BET surface area.

b V_t_ - total pore volume.

c V_mic_-micropore volume.

d AC - coconut shell activated carbon.

Supplementary Figure S2 shows the nitrogen adsorption-desorption isotherms and pore size distributions of BC, BC-KOH_2.5_ and 50MBC-KOH_2.5_. According to the IUPAC classification, the BC isotherm is type IV, whereas those of BC-KOH_2.5_ and 50-MBC-KOH_2.5_ are type I. The BC isotherm has an obvious H_4_ hysteresis loop when *P*/*P*_0_ > 0.4, which indicates the presence of mesopores. The adsorption-desorption isotherms of BC-KOH_2.5_ and 50MBC-KOH_2.5_ are almost parallel, which indicates that micropores are the main porous structure (Alvarez-Torrellas et al., 2016). Supplementary Figure S2B shows that BC has a wide pore size distribution range, whereas the pore size distributions of BC-KOH_2.5_ and 50MBC-KOH_2.5_ are relatively uniform. This indicates that the microporosity increases after KOH activation, which is in agreement with the results reported by Xie et al. (2016). Table 1 shows that the BET specific surface areas of BC, BC-KOH_2.5_, and 50MBC-KOH_2.5_ are 650.8, 1719.0 and 1267.3 m^2^ g^−1^, respectively. The previous researches explained that the increase in the surface area and pore volumes might be caused by the quick release of H_2_ and CH_4_ and the reaction of aromatic condensation when the temperature rose (Zhao et al., 2017; Zhao et al., 2018). As well, when the temperature increased, the formation of more pores and a larger surface area was due to the release of more volatiles from the biomass surface (Cheng and Li, 2018). When the biochar was further activated by KOH, the gaseous CO, CO_2_, H_2_O and H_2_ species generated during activation exit the solid, generating enlarged pore diameters, while an in-situ generated metallic K diffuses into the internal structure (Herath et al., 2020), generating a huge increase in the surface area (1719.0 m^2^ g^−1^). This finding was consistent with the observation of the SEM analysis. The increase in the void volume and decrease in the average pore size indicate that activation produces more micropores and ultra-micropores (Herath et al., 2020). Part of the porous structure collapsed or was covered by iron oxide after magnetization (Dewage et al., 2019; Zhang R et al., 2020) and the BET surface area decreased, but the pore volume and average pore size changed little, which indicates that magnetization did not completely destroy the porous structure.

### 3.4 Magnetic, XRD, Raman and FTIR Analysis

**FIGURE 2:**
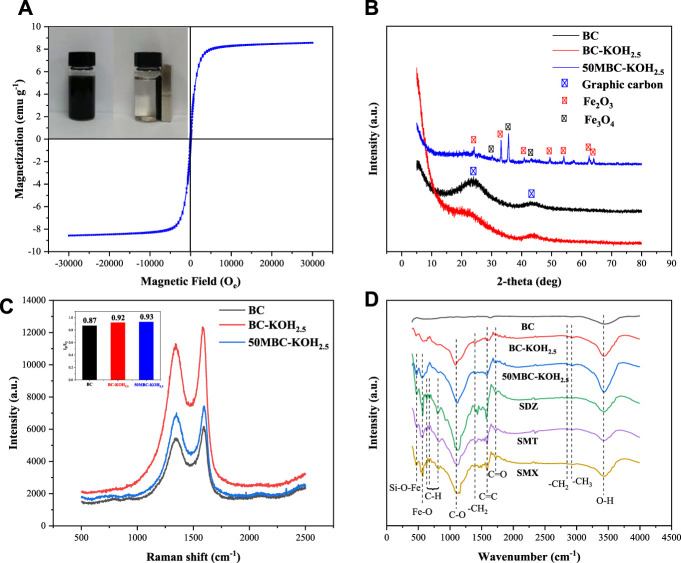
**(A)** Magnetization curve of 50MBC-KOH_2.5_ and photograph of magnetic separation of 50MBC-KOH_2.5_. **(B)** XRD patterns and **(C)** Raman spectra of BC, BC-KOH_2.5_ and 50MBC-KOH_2.5_. **(D)** FTIR spectra of three adsorbents, and of 50MBC-KOH_2.5_ after adsorption of SDZ, SMT and SMX.

Figure 2A shows the hysteresis curves of 50MBC-KOH_2.5_ at room temperature. The saturation magnetization of 50MBC-KOH_2.5_ is 8.6 emu g^−1^. This could be ascribed to the formation of Fe_3_O_4_. It can be seen that the adsorbent shows a slight coercive force and no obvious magnetization lag. This indicates that the prepared 50MBC-KOH_2.5_ has obvious superparamagnetism. Therefore, the spent 50MBC-KOH_2.5_ with adsorbed SAs can be magnetically separated from the solution by an external magnetic field (Yang et al., 2016).

The mineral phases and crystal structures of the three adsorbents were identified by XRD and Raman spectroscopy. As shown in Figure 2B, the XRD pattern of BC has obvious broad peaks at 2*θ* = 24° and 43°. These correspond to the (002) and (101) crystal planes, respectively, indicating the presence of amorphous carbon and a graphitized structure (Wang et al., 2009). There are π^+^-π electron-donor-acceptor (EDA) interactions between the graphitized structure and the aromatic structures of SAs (Xie et al., 2016). After KOH activation, the two broad peaks are weaker. Figure 2B shows that the degree of graphitization decreases, and there is no sharp peak from BC-KOH_2.5_. This indicates that potassium salts and impurities have been completely removed (Qu et al., 2021). The XRD pattern of BC-KOH_2.5_ is consistent with the previous researches (Otowa et al., 1993; Pezoti et al., 2016; Fu et al., 2019; Prasannamedha et al., 2021). The main peaks and corresponding crystal planes are 30.2° (220), 35.4° (311) and 43.2° (400). This pattern corresponds to magnetite (JCPDS file number 19-0629), indicating 50MBC-KOH_2.5_ successfully formed crystalline Fe_3_O_4_ (Zhang et al., 2011). The diffraction peaks at 2*θ* = 24.1°, 33.1°, 35.4°, 40.9°, 49.4°, 54.1°, 62.5° and 64.0° are in good agreement with those of JCPDS card 33-0664, and indicate the formation of crystalline hematite α-Fe_2_O_3_ [(012), (104), (110), (113), (024), (116), (214) and (300)] (Dewage et al., 2019).

The Raman spectrum clearly shows graphitization of 50MBC-KOH_2.5_. The Raman spectra of the three types of biochar are shown in Figure 2C. The peak at 1350 cm^−1^ (D band) is related to disordered sp^2^ hybridization of carbon atoms and the presence of vacancies, impurities, or defects (such as oxygen-containing functional groups). The peak at 1590 cm^−1^ (G band) indicates structural integrity resulting from sp^2^ carbon hybridization (Ahmed et al., 2017). The ratio of the intensity of the D band to that of the G band (*I*_D_/*I*_G_) indicates the degree of graphitization. The *I*_D_/*I*_G_ values of BC, BC-KOH_2.5_, and 50MBC-KOH_2.5_ are 0.87, 0.92 and 0.93, respectively. The decreased graphitization degree after KOH activation may be due to the insertion of potassium vapor into the carbon layer, which could destroy part of the graphitized structure (Xie et al., 2016). The degree of graphitization decreased slightly after magnetization, which indicated that the degree of disorder increases. The *I*_*D*_ */I*_*G*_ value of 50MBC-KOH_2.5_ (0.93) is higher than that of BC-KOH_2.5_ (0.92), suggesting that Fe species may incorporate into the sp^2^ carbon network of 50MBC-KOH_2.5_ biochar and it makes the more defective (Jiang et al., 2017; Liu et al., 2019). Li et al. (2021) reported that it would be due to the formation of heteroatoms and the defects by metal insertion.

The surface functional groups of the three types of biochar and 50MBC-KOH_2.5_ with adsorbed SAs were identified by FTIR, as shown in Figure 2D. For BC, there are few functional groups on the surface, and the 3432 cm^−1^ band is attributed to the O-H stretching vibration, which indicates the presence of hydroxyl groups (Zhang R et al., 2020). However, the number of surface functional groups increases after KOH activation, and a band at 1089 cm^−1^ from the C-O vibration appears. This indicates the presence of hydroxyl and carboxylate groups (Lv et al., 2016). The presence of -OH and C-O indicates that the BC-KOH_2.5_ surface is rich in oxygen-containing functional groups, and the abundance of -OH increased. The possible mechanism for formation of these functional groups: 1) KOH was able to react with active O-containing species to remove the O-containing groups, meanwhile did form several vacancies, followed by some OH^−^ (anions) from KOH that rapidly entered these vacancies and formed new O-containing species (such as C=O, -OH, C-O, -O-C=O and -COOH groups); 2) KOH could also etch carbon fragments to form some vacancies (Niu et al., 2017), which were filled with OH^−^ to form new O-containing species, as expressed by Eq. 12 (Oh et al., 2018); and 3) KOH reacted with biomass and generated a large amount of H_2_O and CO_2_, which further reacted with biomass to form new O-containing species (Chang et al., 2017; Heo and Park, 2018). The increase of oxygen-containing functional groups is consistent with the elemental analysis results. Herath et al. (2020) obtained similar results. A bimodal phenomenon was observed at 2921 and 2845 cm^−1^, which may arise from the tensile vibration of C-H in methyl and methylene. The bands at 1715 and 1586 cm^−1^ correspond to the tensile vibrations of C=O and C=C, respectively (Lahtinen et al., 2014; Yang J et al., 2019). The band at 1391 cm^−1^ corresponds to the bending vibration of -CH_2_ (Chen et al., 2017). After magnetization, two new peaks appear, at 559 and 472 cm^−1^, which are attributed to the Fe-O tensile vibration of Fe_3_O_4_ and the bending vibration of Si-O-Fe (Wan et al., 2020). This indicates successful synthesis of magnetic biochar, and is consistent with the XRD results.

$$OH^{-}\;+\;C\left(vacancies\right)\;\to \;\left(C=O\right)\;+\;\left(-OH\right)\;+\;\left(C-O\right)\;+\;\left(O-C=O\right)\;+\;\left(-COOH\right)$$ (12)

Compared with the 50MBC-KOH_2.5_ spectrum before adsorption, that of 50MBC-KOH_2.5_ with adsorbed SAs shows new peaks in the range 900-600 cm^−1^. These bands are related to the out-of-plane bending vibration of C-H bonds in aromatic compounds (Köseoğlu et al., 2015). In addition, the weakening of the peaks at 3432 and 1089 cm^−1^ indicates that hydrogen bonds are formed between SA molecules and the -OH/-COOH groups of 50MBC-KOH_2.5_ (Zhang X et al., 2020). This indicates that SAs were successfully adsorbed on 50MBC-KOH_2.5_.

### 3.5 Adsorption Kinetics and Isotherms

**FIGURE 3:**
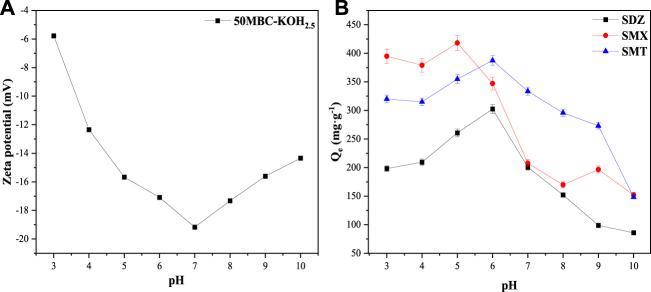
**(A)** Zeta potential of 50MBC-KOH_2.5_ at different pH values and **(B)** effects of solution pH on adsorption capacities for SDZ, SMT and SMX on 50MBC-KOH_2.5_.

The kinetics of adsorption of SDZ, SMT and SMX on 50MBC-KOH_2.5_ are shown in Figure 3A. It can be seen that the adsorption rates of the three SAs in the first 15 min are very fast. The reason may be that there are abundant active adsorption sites on the surface of the magnetic biochar in the initial stage of adsorption. As adsorption progresses, the adsorption sites are gradually occupied, which causes the adsorption to slow down. Because of the decreased number of adsorption sites and the increased electrostatic repulsion between the SA molecules on the surface of the magnetic biochar and the SA molecules in the solution, adsorption equilibrium is finally reached (Patrycja et al., 2019).

As shown in Supplementary Figure S3A, both pseudo-first-order and pseudo-second-order kinetic models fit the rapid adsorption stage well. The adsorption kinetic constants of the pseudo-first-order and pseudo-second-order rate equations for the three sulfonamides are given in Table 2. The *Q*_e_ values obtained with the pseudo-first-order rate equation are different from the experimental values for the SAs, and the correlation coefficients *R* ^2^ are smaller than those for the pseudo-second-order kinetic model. The Chi^2^ and error values for the pseudo-second-order kinetic model are smaller than those for the pseudo-first-order kinetic model. This indicates that the pseudo-second-order kinetic model best describes the process of SA adsorption on 50MBC-KOH_2.5_.

**TABLE 2:** Adsorption kinetics parameters of SAs on 50MBC-KOH_2.5_.

Kinetic models		Pseudo-first-order			Pseudo-second-order		
Adsorbates	Q_e,exp_ (mg·g^−1^)	K_1_ (min^−1^)	Q_e,cal_ (mg·g^−1^)	R^2^	K_2_ (g·mg^−1^·min^−1^)	Q_e,cal_ (mg·g^−1^)	R^2^
SDZ	273.59	0.0053	47.59	0.956	0.0017	270.27	0.993
SMT	365.75	0.0350	114.09	0.921	0.0015	357.14	0.991
SMX	434.94	0.0555	168.22	0.930	0.0012	434.78	0.990

The equilibrium concentrations of the SAs and adsorption capacities are shown in Supplementary Figure S3B. It can be seen that as the initial concentrations of the three SAs increase, the adsorption capacities increase. When the initial concentrations of the SAs are low, the availability of adsorption sites on the 50MBC-KOH_2.5_ surface is very high. When the SA concentration is 10 mg L^−1^, almost 100% of the adsorption sites are occupied. As the initial SA concentration increases, the availability of binding sites decreases and the removal rate decreases.

As shown in Supplementary Figure S3B and Table 3, the experimental data for SAs-50MBC-KOH_2.5_ interactions is more consistent with the Langmuir equation (*R* ^2^ > 0.99), which indicates that the adsorbent surface is rich in active adsorption centers, and adsorption of the three antibiotics follows a monolayer adsorption mechanism (Liu et al., 2019; Zhang X et al., 2020). The maximum adsorption capacity *Q*_m_ obtained by fitting to the Langmuir model is slightly higher than the experimental adsorption capacity *Q*_e_. Although the *R* ^2^ value for the Freundlich equation is smaller than that for the Langmuir equation, the Freundlich equation also fits the experimental data for SDZ and SMT well (*R* ^2^ > 0.9). The fitting with the Freundlich model indicates heterogeneity of the adsorbent surface caused by the presence of Fe_3_O_4_ and Fe_2_O_3_ sites, and indicates that there may be multi-layer complexation (Joanna et al., 2018). The value of *1/n* in the Freundlich equation calculated by the model is between 0 and 1, which indicates that adsorption is disadvantageous (Mayakaduwa et al., 2016). The adsorbents obtained in this study have better adsorption properties. Exploring the KOH and FeCl_3_·6H_2_O dosages enabled preparation of economic adsorbents with excellent adsorption properties.

**TABLE 3:** Adsorption isotherm constants for SAs adsorption onto 50MBC-KOH_2.5_ at 298 K.

Isotherm models		Langmuir			Freundlich		
Adsorbates	Q_e,exp_ (mg·g^−1^)	Q_m_ (mg·g^−1^)	K_L_ (L·mg ^−1^)	R^2^	K_F_ (mg·g^−1^) (L·mg ^−1^) ^1/n^	1/n	R^2^
SDZ	289.17	294.12	0.330	0.997	118.180	0.245	0.962
SMT	391.39	400.00	0.333	0.997	160.774	0.217	0.955
SMX	453.34	454.55	0.579	0.999	284.690	0.111	0.935

### 3.6 Influences of Initial pH and Co-existing Ions on SAs Removal by 50MBC-KOH_2.5_

The Zeta potentials and adsorption capacities of 50MBC-KOH_2.5_ for the three SAs at different pH are shown in Figures 3A,B, respectively. Supplementary Table S5 and Supplementary Figure S4 show the distributions of SA amphoteric molecular species at different pH values. It can be seen that 50MBC-KOH_2.5_ is negatively charged in the pH range selected for the experiments. At low pH values, SA^+^ is present and π^+^-π EDA interactions are the main mechanism because sulfonamides are strong π receptors, and the adsorbents contain functional groups such as -OH, C=C and -COOH, which can be used as powerful π-electron donors (Nam et al., 2015; Ahmed et al., 2017). There is also electrostatic attraction between the negatively charged adsorbent and SA^+^, therefore the adsorption capacity at low pH values is relatively high. With increasing pH, a small amount of charge-assisted hydrogen-bonding (CAHB) occurs between SA^−^ and -OH (Chen Z. et al., 2019). Even if there is electrostatic repulsion between the adsorbent and SA^−^, the adsorption capacity reaches the maximum. This indicates that electrostatic interaction is only part of the adsorption mechanism (Cheng et al., 2020). When the pH increases further, SA^−^ species gradually becomes dominant, and electrostatic repulsion between the adsorbent and SA^−^ increases, which results in a significant decrease in the adsorption capacity (Azhar et al., 2017). Many adsorption mechanisms such as π^+^-π EDA, CAHB, electrostatic, Lewis acid-base interactions, pore filling, van der Waals forces and hydrophobic interactions are involved in the removal of SAs (Tian et al., 2019). However, these mechanisms may have different contribution ratios at different pH (Chen X et al., 2019). The differences among the adsorption capacities for different sulfonamides are therefore caused by differences among their own properties and the different contribution ratios of various mechanisms.

**FIGURE 4:**
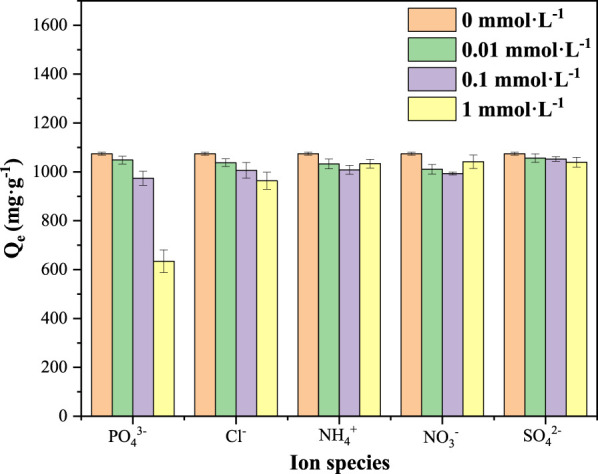
Effect of ion strength on 50MBC-KOH_2.5_ adsorption of SAs in actual water.

Given the fact that the potential matrix species in actual aqueous environment may influence the removal of pollutants, the effect of ion species (PO_4_^3−^, Cl^−^, NH_4_^+^, NO_3_^−^ and SO_4_^2−^) and strength (0–1 mmol L^−1^) on the adsorption of SAs by 50MBC-KOH_2.5_ were further investigated. As shown in Figure 4, except for PO_4_^3−^, the other four ions had little effect on the total adsorption capacity of SAs. When the concentration of PO_4_^3−^was in the range of 0.01–0.1 mmol L^−1^, the total adsorption capacity of SAs was slightly decreased (<10%). However, the high PO_4_^3−^ concentration (1 mmol L^−1^) exhibited a pronounced inhibitory effect (41%) on 50MBC-KOH_2.5_ adsorption of SAs, which could be attributed to the reaction between PO_4_^3−^ and the iron oxides on 50MBC-KOH_2.5_ (Zhang R et al., 2020). Overall, the inhibition of the presence of anions for SAs adsorption on 50MBC-KOH_2.5_ can be explained by effect of competition effect for actives sites or the acidity of functional groups (e.g., −COOH) (Wang et al., 2019; Dai et al., 2020; Zhang et al., 2021).

### 3.7 Adsorption Mechanism Analysis

**FIGURE 5:**
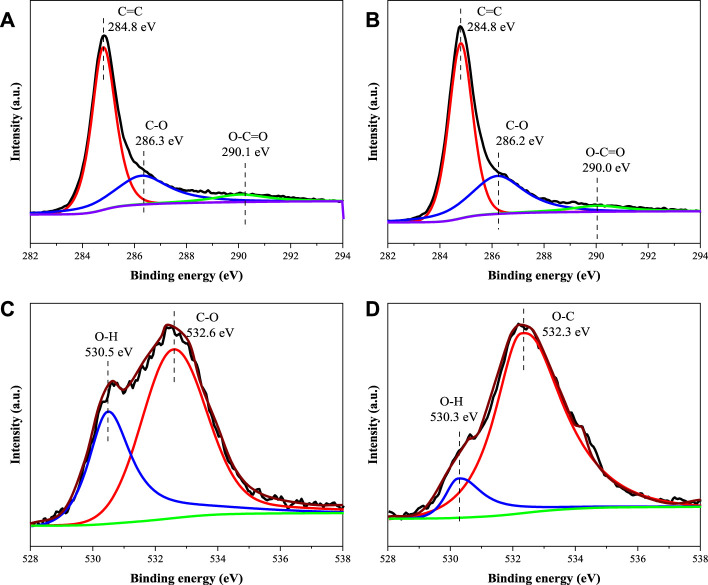
High-resolution XPS spectra of C1s of **(A)** fresh 50MBC-KOH_2.5_ **(B)** spent 50MBC-KOH_2.5_ and of O1s of **(C)** fresh 50MBC-KOH_2.5_ **(D)** spent 50MBC-KOH_2.5_.

The above results show that the process of SA adsorption on 50MBC-KOH_2.5_ includes both physical adsorption and chemical adsorption. As shown in Supplementary Figure S5, the π system on the two aromatic rings of sulfonamides is electron deficient and has a strong electron-absorbing ability. The high electronegativity of O atoms leads to stability via electron resonance and further reduces the π-electron cloud density of aromatic rings (Chathuri et al., 2017). Due to the amino functional group can provide lone-pair electrons to the aromatic ring, it can also be used as a π receptor (Ahmed et al., 2017). The surface of the adsorbent has a graphitized structure and contains π-electron-donor functional groups such as -OH, C=C and -COOH, so there are π^+^-π EDA interactions between the adsorbent and the SAs. When both the adsorbent and SAs are negatively charged, CAHB can occur because of the presence of polar functional groups (Chathuri et al., 2017). In the presence of neutral sulfonamide molecules, there is a strong Lewis acid-base interaction between the basic groups of SAs and -COOH and -OH on the adsorbent surface (Ahmed et al., 2017). Raman and IR spectra confirmed the above results. In addition, because of the negative charge on the adsorbent surface and the presence of charged SA species, electrostatic interaction is also an important adsorption mechanism. The XPS peak deconvolution for C1s and O1s of the 50MBC-KOH_2.5_ before and after SAs adsorption were showed in Figure 5. For the region of C1s, the peaks at 284.8, 286.3 and 290.1 eV were associated to C=C, C-O and O-C=O (Yang et al., 2016). The O1s spectra could be divided into two peaks at 530.5 eV (O-H) and 532.6 eV (O-C) (Zhang et al., 2020). Summary of the peak area ratio for C1s and O1s of fresh and spent 50MBC-KOH_2.5_ were listed in Supplementary Table S6. After SAs adsorption, the oxygen-rich functional groups, such as O-C=O and O-H, decreased from 6.35% to 4.66%, and from 5.18% to 2.08%, respectively, which indicated that the -OH and -COOH groups on the surface of 50MBC-KOH_2.5_ played an essential role in the SAs adsorption via hydrogen bonding or Lewis acid-base interaction, which was in accordance to the FTIR results. A high temperature and KOH activation provide abundant active adsorption sites for the above interactions by increasing the specific surface area and porosity of 50MBC-KOH_2.5_, therefore the prepared adsorbents have good adsorption properties (Xie et al., 2016).

### 3.8 Recycling Capacity

**FIGURE 6:**
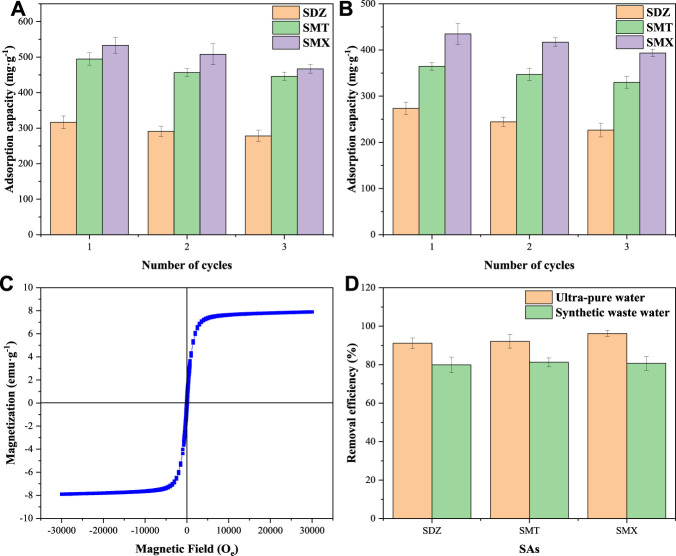
The reusability tests for BC-KOH_2.5_ **(A)** and 50MBC-KOH_2.5_ **(B)**. **(C)** The magnetization curve of 50MBC-KOH_2.5_ after three cycles. **(D)** Efficiencies of SA (50 mg L^−1^) removal by adsorption on 50MBC-KOH_2.5_ from ultra-pure water and synthetic wastewater in 24 h at pH 5.0.

The regeneration capacity is an important indicator in evaluating the cost effectiveness of adsorbents. The regeneration process can reduce the risk of secondary pollution by recovering pollutants (C et al., 2018). In the desorption process, after treatment with 1.0 mol L^−1^ NaOH solution, most of the adsorbed SAs were desorbed from BC-KOH_2.5_ and 50MBC-KOH_2.5_. As shown in Figures 6A,B, after three consecutive adsorption-desorption cycles, the total adsorption capacities for SAs of BC-KOH_2.5_ and 50MBC-KOH_2.5_ decreased by 9.23 and 12.80%, respectively. High adsorption capacities were still obtained for two adsorbents. The hysteresis curve of the adsorbent at this time was examined. As shown in Figure 6C, the saturation magnetization after three cycles decreased from 8.6 to 7.9 emu g^−1^ (less than 10%), the leaching of total dissolved Fe from 50MBC-KOH_2.5_ was low, indicating the stability of magnetic properties of the adsorbent (Yan et al., 2022).

### 3.9 SAs Removal From Synthetic Wastewater

Figure 6D shows the removal efficiencies of SAs by 50MBC-KOH_2.5_ in ultra-pure water and synthetic wastewater. As shown in the figure, more than 90% of the SAs can be removed from ultra-pure water within 24 h. In the synthetic wastewater, the average removal rates for SDZ, SMT and SMX were 78.70, 82.18 and 86.29%, respectively; these are similar to previously reported values (Ahmed et al., 2018). This shows that the presence of other compounds in the synthetic wastewater inhibits the adsorption of SAs on 50MBC-KOH_2.5_. This inhibition may be caused by competition between the SAs and other compounds in the wastewater for the active sites and pores (Premarathna et al., 2019). Nevertheless, about 80% of SAs can be removed from wastewater. In addition, the concentrations of SAs in actual wastewater are relatively low, and even with the influence of other compounds, 50MBC-KOH_2.5_ can effectively remove SAs in wastewater.

## 4 Conclusion

KOH-activated and FeCl_3_-magnetized biochar was developed as a recyclable adsorbent for sorptive removal of antibiotics from wastewater. The maximum adsorption capacities for SDZ, SMT and SMX were 294.12, 400.00 and 454.55 mg g^−1^, respectively. The adsorption kinetics of the three kinds of SAs fitted the pseudo-second-order rate equation and the adsorption isotherms conformed to the Langmuir model. The adsorption of SAs by 50MBC-KOH_2.5_ is pH dependent, and the removal effect was better under acidic and slightly acidic conditions. In addition, this carbon material has good recovery and regeneration abilities, and can effectively remove SAs from wastewater. Mechanistic analysis showed that π^+^-π EDA, CAHB, electrostatic and Lewis acid-base interactions may be the main reasons for adsorption of SAs on 50MBC-KOH_2.5_.

## Data Availability

The original contributions presented in the study are included in the article/Supplementary Material, further inquiries can be directed to the corresponding authors.
