# Supplementary material for: Adsorption of Sulfonamides in Aqueous Solution on Reusable Coconut-Shell Biochar Modified by Alkaline Activation and Magnetization
Source: Front Chem. 2022 Jan 21;9:814647. doi: 10.3389/fchem.2021.814647 (PMC8813774; doi:10.3389/fchem.2021.814647)
Supplement: Supplementary file 1 [file DataSheet1.doc]

**Adsorption of sulfonamides in aqueous solution on reusable coconut**-**shell biochar modified by alkaline activation and magnetization**

Ying Sun ^a,b,1^, Lili Zheng ^a,c,1^, Xiaoyan Zheng ^a,c^, Dao Xiao ^a,c^, Yang Yang ^a,c^, Zhengke Zhang ^b^, Shenwan Wang ^a,b^, Binling Ai ^a,c^,*, Zhanwu Sheng ^a,c^,*

^a^ Haikou Experimental Station, Chinese Academy of Tropical Agricultural Sciences, Haikou 571101, China

^b^ College of Food Science and Engineering, Hainan University, Haikou 570228, China

**^c^** Haikou Key Laboratory of Banana Biology, Haikou 571101, China

^1^These authors contributed equally.

*Corresponding authors:

Binling Ai: aibinling@catas.cn

Zhanwu Sheng: shengz@catas.cn

**Table S1** Comparison of maximum adsorption for sulfonamide antibiotics on various activated carbon materials reported in previous studies.

| Adsorbents | Adsorbate | Q_m_ (mg·g^-1^) | References |
| --- | --- | --- | --- |
| Coal-based [powdered activated carbon](https://www.sciencedirect.com/topics/engineering/powdered-activated-carbon) | SMX | 91.5 | (Luo et al., 2021) |
| Wood-based granular activated carbon | SMX | 258.7 |  |
| Coconut shell-based granular activated carbon | SMX | 184.7 |  |
| Olive pomace-based [powdered activated carbon](https://www.sciencedirect.com/topics/engineering/powdered-activated-carbon) | SDZ | 66.2 | (Aslan and Şirazi, 2020) |
| Granular activated carbon | SMR | 165.7 | (Yao et al., 2019) |
| Activated carbon modified by FeCl_3_ | SMT | 17.2 | (Liu et al., 2017) |
| Coconut shell-derived pulverized activated carbon | SMX | 12.7 | (Ji et al., 2010) |
| Bituminous coal-derived pulverized activated carbon | SMX | 30.4 |  |
| Powdered activated carbon from pine tree | SMX | 131.7 | (Tonucci et al., 2015) |
| Activated carbons derived from cottonseed husks | SA | 146.1 | (Xu et al., 2021) |
| Bagasse-derived activated carbon | SMX | 294.1 | (Hu et al., 2021) |
| Coconut shell biochar | SDZ | 273.6 | This work |
|  | SMT | 365.8 |  |
|  | SMX | 434.9 |  |

**Table S2** Summary of various modified adsorbents for sulfonamides removal.

| Adsorbents | Surface area (m^2^·g^-1^) | Total pore volume (cm^3^·g^-1^) | Adsorbate | Q_m_ (mg·g^-1^) | References |
| --- | --- | --- | --- | --- | --- |
| [Corn stalk](https://www.sciencedirect.com/topics/engineering/corn-stalk) biochar | 34.9 | 1.334 | SPY | 76.99 | (Tang et al., 2021) |
| Single-walled CNTs | 541 | - | STZ | 239 | (Liu et al., 2016) |
| Reduced graphene oxides | 331 | - | STZ | 143 |  |
| Multiwalled CNTs | 117 | - | STZ | 57.7 |  |
| Graphite | 7 | - | STZ | 6.17 |  |
| Rice straw  biochar | 5.76 | 0.013 | SMX | 6.75 | (Sun et al., 2016) |
| Sulfonated graphene oxide | - | - | SMX | 312.28 | (Liu et al., 2021) |
|  |  |  | SPD | 161.89 |  |
| Pinus radiata [sawdust](https://www.sciencedirect.com/topics/earth-and-planetary-sciences/sawdust) biochar | 125.8 | 0.14 | SMX | 13.83 | (Reguyal et al., 2017) |
| Graphene | 246 | 0.632 | SMX | 46.14 | (Luo et al., 2021) |
| Coal-based [powdered activated carbon](https://www.sciencedirect.com/topics/engineering/powdered-activated-carbon) | 378 | 0.382 | SMX | 91.51 |  |
| Wood-based granular activated carbon | 963 | 0.578 | SMX | 258.7 |  |
| Coconut shell-based granular activated carbon | 936 | 0.506 | SMX | 184.7 |  |
| Metal-organic frameworks | - | - | SCP | 384 | (Azhar et al., 2016) |
| Faujasite zeolite Y | 852 | 0.62 | SCP | 280 | (Braschi et al., 2010) |
| Wheat stalk biochar | 155.426 | 0.190 | SDZ | 51.724 | (Yan et al., 2022) |
| Tea waste biochar | 648.415 | 0.370 | SDZ | 77.52 | (He et al., 2021) |
| Sugarcane bagasse biochar | 1099 | 1.239 | SMX | 400 | (Prasannamedha et al., 2021) |
| Biological sludge and iron sludge | 1385.5 | 0.63 | SMT | 178.6 | (Wan et al., 2020) |
|  |  |  | SA | 63.7 |  |
|  |  |  | SDZ | 140.9 |  |
|  |  |  | SMX | 181.8 |  |
| Coconut shell biochar | 1267.3 | 0.7095 | SDZ | 273.59 | This work |
|  |  |  | SMT | 365.75 |  |
|  |  |  | SMX | 434.94 |  |

**Table S3** The UPLC elution program.

| Time (min) | Flow rate (mL·min^-1^) | % A | %B |
| --- | --- | --- | --- |
| 0 | 0.3 | 90 | 10 |
| 5 | 0.3 | 40 | 60 |
| 5.1 | 0.3 | 90 | 10 |
| 8 | 0.3 | 90 | 10 |

**Table S4** Experimental adsorption capacity (Q_e_, mg·g^-1^) of carbonaceous materials for SAs.

| Adsorbates | BC | BC-KOH_2.5_ | 50MBC-KOH_2.5_ |
| --- | --- | --- | --- |
| SDZ | 41.04 | 328.31 | 273.59 |
| SMT | 73.15 | 475.48 | 365.75 |
| SMX | 78.29 | 530.63 | 434.94 |
| Sum | 192.48 | 1334.42 | 1074.28 |

**Table S5** Structure and physicochemical properties of different sulfonamides.

| Molecular structure | Name | Molecular weight | R | pK_a1_ | pK_a2_ |
| --- | --- | --- | --- | --- | --- |
| 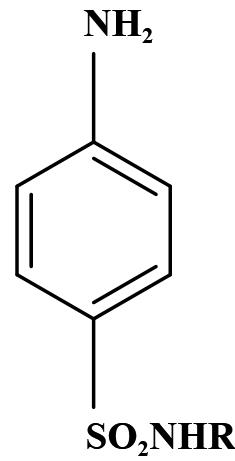 | SDZ | 264.30 | 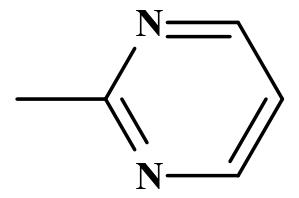 | 1.6 | 6.5 |
|  | SMT | 278.33 | 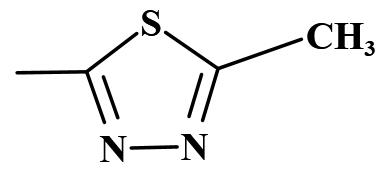 | 2.7 | 7.7 |
|  | SMX | 310.33 | 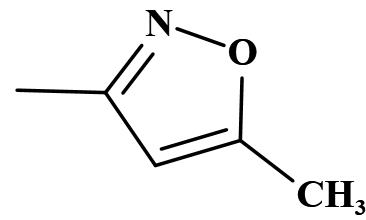 | 1.6 | 5.7 |

**Table S6** Summary of the peak area ratio of C 1s and O 1s of 50MBC-KOH_2.5_ before and after adsorption.

| Binding energy (eV) | 284.8 | 286.3 | 290.1 | 530.5 | 532.6 |
| --- | --- | --- | --- | --- | --- |
| Functional groups | C=C | C-O | O-C=O | O-H | O-C |
| Before adsorption | 53.13% | 24.57% | 6.35% | 5.18% | 9.18% |
| After adsorption | 43.93% | 27.38% | 4.66% | 2.08% | 15.15% |

**Fig. S1.** (a) Comparison of total SAs adsorption capacity on various agriculture waste-derived biochar. (b) Sum of adsorption capacity of three kinds of sulfonamides antibiotics at different mass ratios of KOH to BC-400 (1:1,1:1.5,1:2,1:2.5,1:3,1:3.5,1:4,1:4.5,1:5,1:5.5) and different FeCl_3_·6H_2_O (25、50、75、100 mmol·L^−1^) concentrations.

**Fig. S2.** (a) N_2_ adsorption-desorption isotherms of BC、BC-KOH_2.5_ and 50MBC-KOH_2.5_; (b) pore size distributions of BC、BC-KOH_2.5_ and 50MBC-KOH_2.5_.

**Fig. S3.** Adsorption kinetics and adsorption isotherms for SDZ, SMT, and SMX on 50MBC-KOH_2.5_.


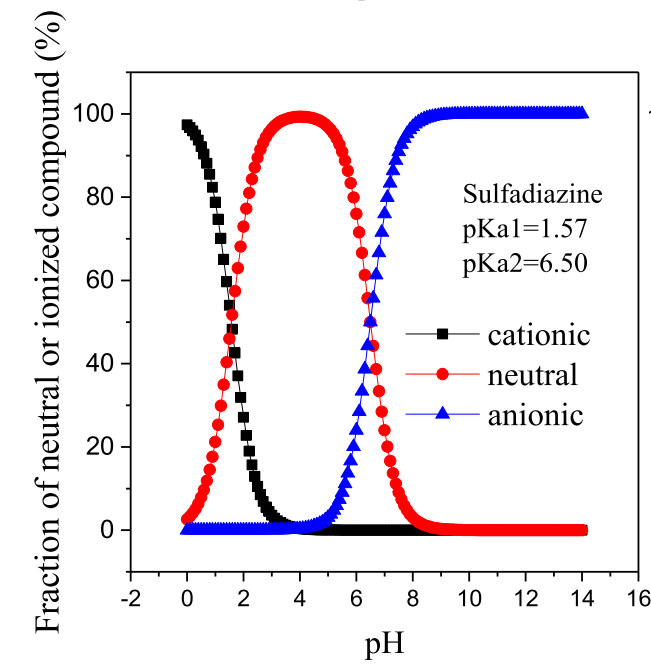

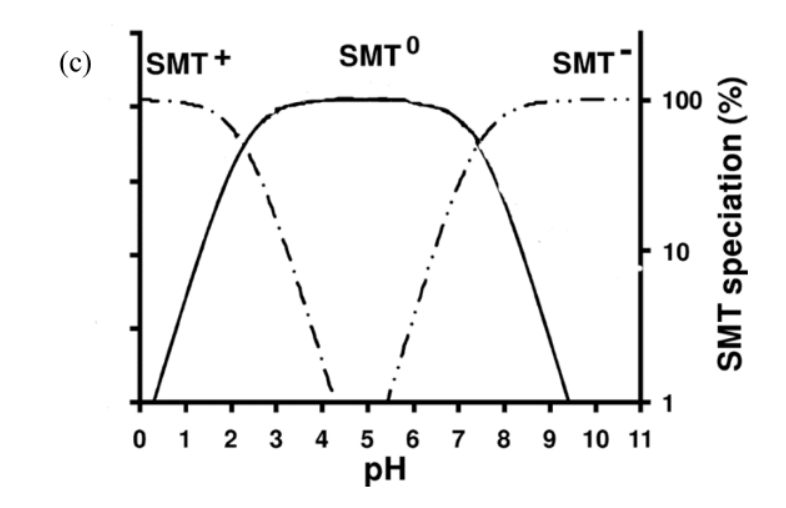

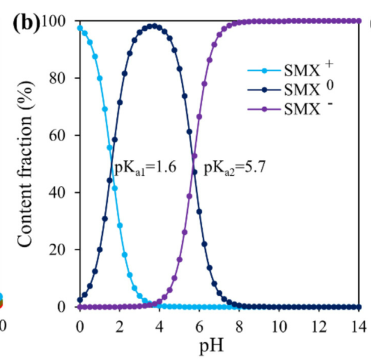


**Fig. S4.** The chemical speciation of (a) sulfadiazine modified from B et al. (B et al., 2020); (b) sulfamethazine modified from Teixidó et al. (Teixidó et al., 2011), and (c) sulfamethoxazole taken from Xie et al. (Xie et al., 2020).


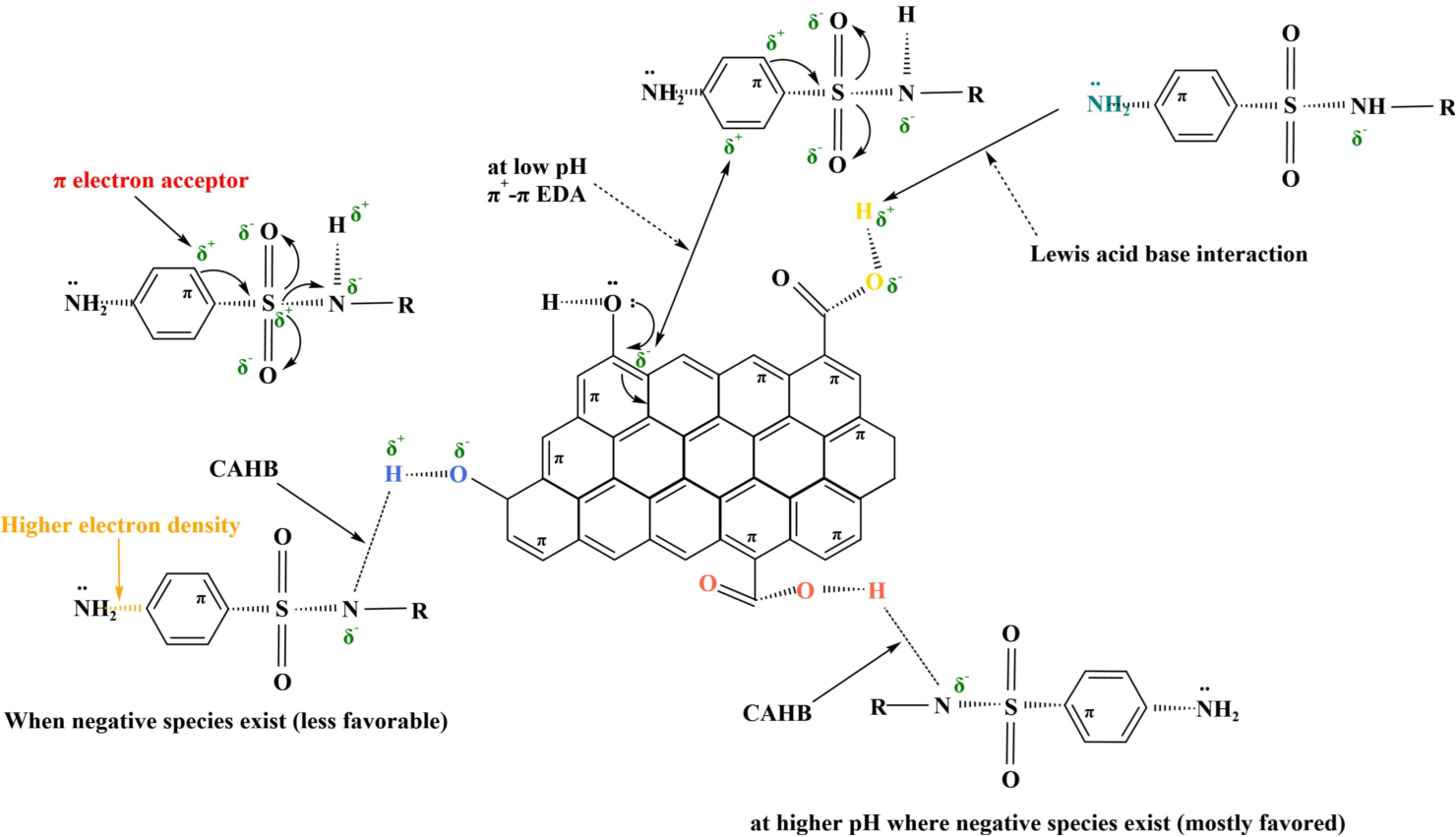


**Fig. S5.** Proposed sorption mechanism for SAs on 50MBC-KOH_2.5_.
